# Spinal motor neurons are regenerated after mechanical lesion and genetic ablation in larval zebrafish

**DOI:** 10.1242/dev.129155

**Published:** 2016-05-01

**Authors:** Jochen Ohnmacht, Yujie Yang, Gianna W. Maurer, Antón Barreiro-Iglesias, Themistoklis M. Tsarouchas, Daniel Wehner, Dirk Sieger, Catherina G. Becker, Thomas Becker

**Affiliations:** Centre for Neuroregeneration, The University of Edinburgh, 49 Little France Crescent, Edinburgh EH16 4SB, UK

**Keywords:** Dopamine, Macrophage, Microglia, Nitroreductase, Hb9, Olig2, Sox10

## Abstract

In adult zebrafish, relatively quiescent progenitor cells show lesion-induced generation of motor neurons. Developmental motor neuron generation from the spinal motor neuron progenitor domain (pMN) sharply declines at 48 hours post-fertilisation (hpf). After that, mostly oligodendrocytes are generated from the same domain. We demonstrate here that within 48 h of a spinal lesion or specific genetic ablation of motor neurons at 72 hpf, the pMN domain reverts to motor neuron generation at the expense of oligodendrogenesis. By contrast, generation of dorsal Pax2-positive interneurons was not altered. Larval motor neuron regeneration can be boosted by dopaminergic drugs, similar to adult regeneration. We use larval lesions to show that pharmacological suppression of the cellular response of the innate immune system inhibits motor neuron regeneration. Hence, we have established a rapid larval regeneration paradigm. Either mechanical lesions or motor neuron ablation is sufficient to reveal a high degree of developmental flexibility of pMN progenitor cells. In addition, we show an important influence of the immune system on motor neuron regeneration from these progenitor cells.

## Abstract (precis)

**Summary:** Regeneration of spinal motor neurons following mechanical lesion or genetic ablation occurs at the expense of oligodendrogenesis and is promoted by the innate immune system.

## INTRODUCTION

In contrast to mammals, adult zebrafish are capable of regenerating neurons in the central nervous system (CNS), including the spinal cord (Grandel and Brand, 2013; Goldman, 2014; Becker and Becker, 2015; Than-Trong and Bally-Cuif, 2015). To understand these differences, it is important to elucidate the signals and mechanisms leading to successful CNS regeneration in fish. In adult zebrafish, a lesion to the spinal cord induces ependymo-radial glial cells (ERGs) to proliferate and, subsequently, distinct ERG domains give rise to different types of neurons (Reimer et al., 2008; Goldshmit et al., 2012; Kuscha et al., 2012a,b). For example, *olig2*-expressing ERGs in the ventromedial aspect of the spinal cord generate new motor neurons after a lesion (Reimer et al., 2008).

Motor neuron-generating adult ERGs are likely to be derived from embryonic motor neuron progenitor (pMN) cells. The spinal pMN domain thus transitions from a motor neuron generating program during early development [up to 48 hours post-fertilisation (hpf)] (Reimer et al., 2013) to generation of oligodendrocytes (Kirby et al., 2006; Kim et al., 2008; Czopka et al., 2013) and eventually, to relative quiescence at the adult stage (Reimer et al., 2008).

A mechanical lesion in adults reinitiates the program for motor neuron generation (Reimer et al., 2008). This is positively regulated by Hedgehog (Reimer et al., 2009), dopamine signalling acting on the Hedgehog pathway (Reimer et al., 2013) and serotonin (Barreiro-Iglesias et al., 2015). To explore the plasticity of spinal progenitors, we asked whether motor neuron regeneration can be triggered when their developmental generation has been completed but cells in the pMN domain are still proliferating and generate oligodendrocytes, and if so, whether progenitors react to signals similar to those during adult regeneration.

The immune system probably plays an important role in regeneration. For example, there is a strong microglia/macrophage reaction to a spinal lesion (Becker and Becker, 2001) and activation of microglia/macrophages alone is sufficient to trigger neuronal regeneration in the adult zebrafish telencephalon (Kyritsis et al., 2012). This suggests that signalling of microglia/macrophages to progenitor cells occurs.

However, with mechanical lesions, it is difficult to dissociate the effects of extrinsic signals from those of injuring the intricate radial processes of the progenitors themselves. For example, it has been shown that the stem cell potential of astrocytes in mammals differs following mechanical lesion versus cell ablation or a chronic disease state (Sirko et al., 2013). Therefore, we use a genetic strategy (Curado et al., 2008) to selectively ablate motor neurons in order to determine whether this loss is sufficient to trigger motor neuron regeneration in the larval spinal cord.

We find that a mechanical lesion of the larval spinal cord leads to regeneration of motor neurons close to the spinal lesion site and that this can be enhanced with a dopamine agonist, similar to adult regeneration. A macrophage/microglial reaction promotes motor neuron regeneration. Cell type-specific ablation is sufficient to induce motor neuron regeneration. Hence, motor neuron regeneration can be studied in larvae, as it replicates some features of adult regeneration. Motor neuron regeneration can be dissociated from mechanical lesion of progenitors and is promoted by the innate immune system.

## RESULTS

### Larval lesion induces local regeneration of motor neurons

**Fig. 1.**
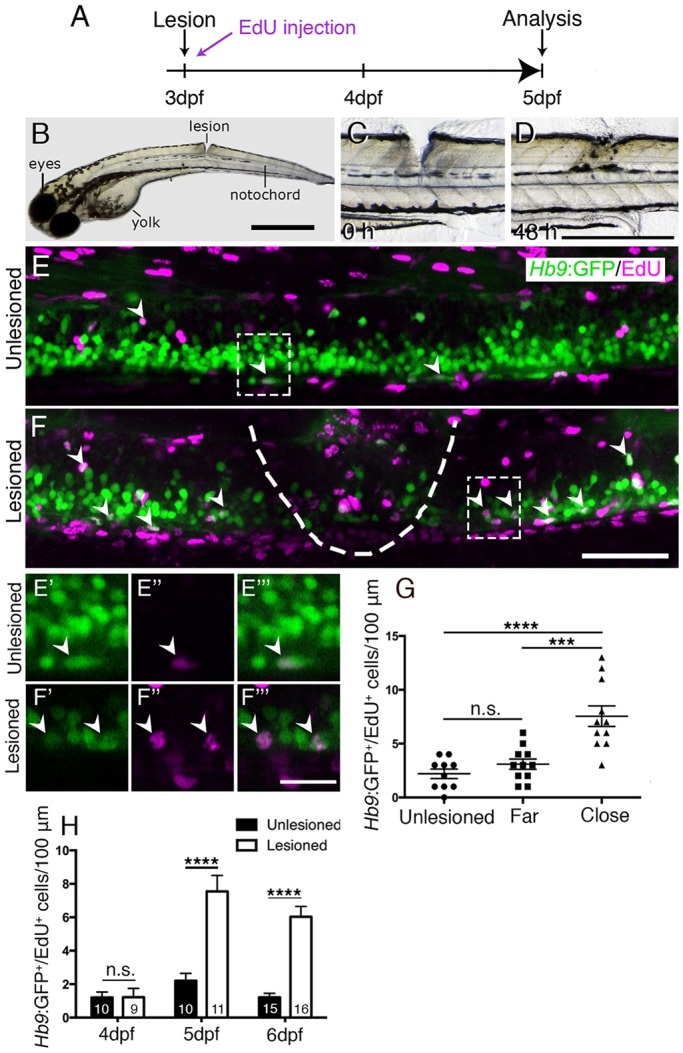
**A mechanical lesion to the spinal cord heals within 48 h and leads to motor neuron regeneration close to the lesion site.** (A) Time line for experiments. (B) A zebrafish larva with a lesion in the dorsal trunk area, leaving the notochord intact at 3 dpf. (C,D) The same larva imaged at 0 and 48 h post lesion shows closure of the wound. (E,F) The lesion area is outlined with double-labelled *Hb9:*GFP^+^/EdU^+^ neurons indicated by arrowheads. A lesion leads to increased numbers of *Hb9:*GFP^+^/EdU^+^ double-labelled motor neurons (compare E and F). E′ to F‴ show higher magnifications of areas boxed in E and F, respectively, in single optical sections indicating double labelling. (G) Motor neurons are regenerated close to (<50 µm rostral and caudal), but not far from (100 µm>×>50 µm rostral and caudal) the lesion site (one-way ANOVA with Bonferroni's multiple comparisons test, ***P<0.001, ****P<0.0001; unlesioned, *n*=10; far, *n*=11; close, *n*=11). (H) Time line of the increase in the number of EdU-labelled motor neurons (*t*-test, ****P<0.0001). Values are means±s.e.m. Lateral views of larvae are shown (rostral is left; dorsal is up). Scale bars: 100 µm in B; 500 µm in D for C,D; 50 µm in F for E,F; 15 µm in F‴ for E′-F‴.

Embryonic motor neuron generation from the pMN domain largely ceases by 48-51 hpf (Reimer et al., 2013), whereas oligodendrogenesis from these progenitors continues for weeks (Park et al., 2007). To determine whether pMN progenitors can switch to generating motor neurons during oligodendrogenesis, we inflicted a mechanical lesion to the spinal cord at 3 days post-fertilisation (dpf), leaving the notochord and major blood vessels intact (Fig. 1A-C). Such a wound closes quickly (within 48 h; Fig. 1D) and larvae recover swimming capability within 2 days post-lesion, as measured by distance moved after tail touch (Fig. S1). This indicates that functional regeneration is extremely quick at the larval stage.

To label newly generated motor neurons, we applied the DNA base analogue and proliferation marker EdU to *Hb9:*GFP (also known as *mnx1:*GFP) transgenic animals directly after the lesion at 3 dpf and counted double labelled neurons at 4, 5 and 6 dpf. No increase in the number of *Hb9:*GFP^+^ neurons that incorporated EdU, which would have been newly generated after the lesion, was observed at 4 dpf. However, at both 5 and 6 dpf, the number of *Hb9:*GFP^+^/EdU^+^ cells was strongly increased by up to 400% close to (within 50 µm), but not far from (within 50-100 µm) the lesion site, compared with unlesioned controls (Fig. 1E-H). To test whether regenerative neurogenesis would also occur at later stages, we shifted the injury paradigm to 5 dpf, when larvae had become fully behaving predators (Semmelhack et al., 2014; Bianco and Engert, 2015). Analysis of *Hb9:*GFP^+^/EdU^+^ neurons at 7 dpf indicated a 257% increase in the number of new motor neurons, comparable to the 3 to 5 dpf standard protocol (Fig. S2). This supports the presence of local lesion-induced signals that lead to motor neuron regeneration within 48 h of the lesion.

**Fig. 2.**
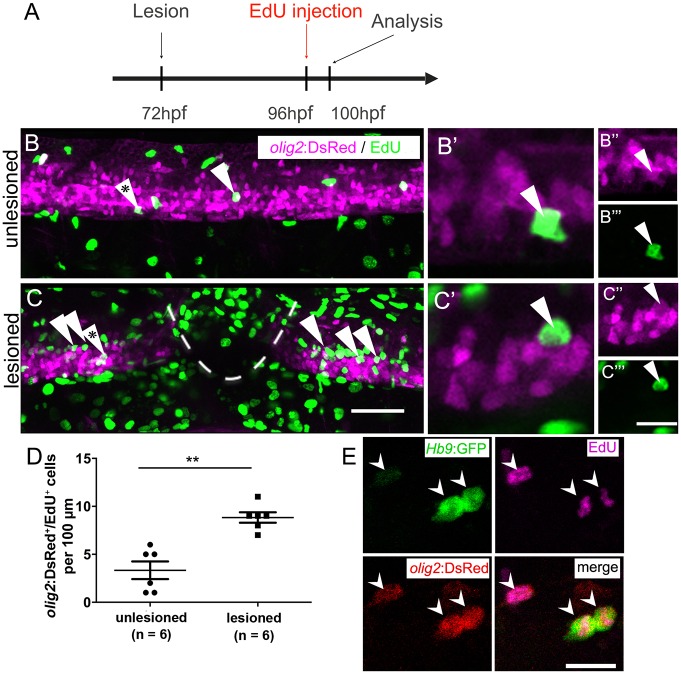
**After a lesion, the pMN domain shows increased proliferation and gives rise to motor neurons.** (A) Time line of the experiment. (B,C) *olig2*:DsRed^+^ cells (arrowheads) in the pMN domain that incorporated EdU within the last 4 h. (B′-C‴) Higher magnifications of single optical sections of the cells indicated by asterisks in B and C, respectively, showing double labelling. (D) The number of proliferating cells in the pMN domain is significantly increased in the vicinity of the lesion site (Mann–Whitney test; **P=0.0049). (E) In *Hb9:*GFP and *olig2*:DsRed double-transgenic larvae (lesion: 3 dpf; analysis: 5 dpf), newly generated motor neurons (*Hb9:*GFP^+^/EdU^+^) that retain DsRed protein are indicated by arrowheads. Lateral views are shown; rostral is left, dorsal is up. Values are means±s.e.m. Scale bars: 50 µm in C for B,C; 20 µm in C‴ for B″-C‴ and 10 µm for B′,C′; 15 µm in E.

To determine whether larval pMN progenitor cells react to a lesion with increased proliferation, we injected EdU into *olig2*:DsRed transgenic larvae, in which pMN progenitors and motor neurons contain DsRed protein. To label acutely proliferating cells in the pMN domain, we chose a paradigm in which larvae were lesioned at 3 dpf and EdU was injected at 4 dpf with a subsequent survival time of 4 h. This indicated a 165% increase in the number of *olig2*:DsRed^+^/EdU^+^ cells (Fig. 2A-D). Hence, proliferation in the pMN domain was increased after a lesion.

To assess whether newly generated motor neurons in larvae were derived from the *olig2*-expressing cells, we used EdU labelling in *Hb9*:GFP; *olig2*:DsRed double transgenic larvae. If newly generated motor neurons are derived from these progenitors, they retain the DsRed protein, which is expressed in progenitors under the regulatory sequences for *olig2*. Thus DsRed protein acts as short-term lineage tracer. Indeed, all newly generated motor neurons (*Hb9*:GFP^+^/EdU^+^; 23 neurons in 4 animals) were also positive for DsRed (Fig. 2E). This suggests that after a lesion, newly generated motor neurons originate from *olig2*-expressing pMN progenitor cells.

**Fig. 3.**
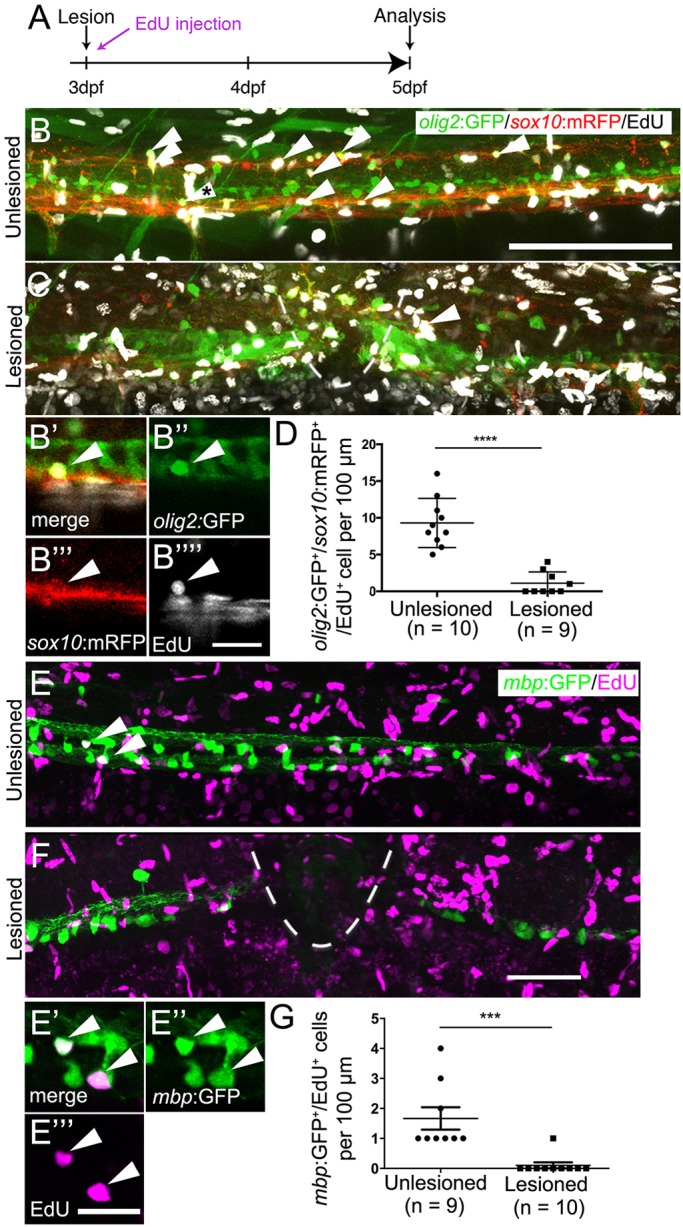
**Oligodendrocyte generation is reduced after a spinal lesion.** (A) Time line of the experiment. (B,C) Newly generated oligodendrocytes and their precursors, triple labelled by *olig2*:GFP, *sox10:*mRFP and EdU (arrowheads), are reduced in number after lesion. (B′-B″″) A triple-labelled cell (indicated with asterisk in B) in a single optical section at higher magnification. (D) The number of triple-labelled cells is reduced (Student's *t*-test, ****P<0.0001). (E) *mbp*:GFP^+^ oligodendrocytes incorporate EdU (indicated by arrowheads) in unlesioned larvae. (E′-E‴) Two double-labelled neurons indicated in E at higher magnification in a single optical section. (F) Fewer double-labelled cells are observed after a lesion. (G) The number of new oligodendrocytes is significantly reduced after a lesion (Mann–Whitney *U*-test; ***P=0.0005). Lateral views are shown; rostral is left, dorsal is up. The lesion site is indicated by a dashed line. Values are means±s.e.m. Scale bars: 100 µm in B for B,C; 20 µm in B″″ for B′-B″″; 50 µm in F for E,F; 20 µm in E‴.

To determine whether generation of oligodendrocytes, which are derived from the same progenitor domain (Kucenas et al., 2008; Czopka et al., 2013), was altered by the lesion, we assessed numbers of *olig2*:GFP/*sox10*:mRFP/EdU triple-labelled cells, representing newly generated oligodendrocytes and their precursors (Kirby et al., 2006), at 5 dpf, after a lesion at 3 dpf. These cells were present in unlesioned larvae, confirming previous evidence for continuous generation of oligodendrocytes in unlesioned larvae (Kucenas et al., 2008; Czopka et al., 2013), and reduced in number by 88% after a lesion (Fig. 3A-D). We assessed the number of newly generated differentiated oligodendrocytes in the *mbp*:GFP (Almeida et al., 2011) transgenic line, in which oligodendrocytes are labelled under the regulatory sequences of the *myelin basic protein a* (*mbpa*) gene. The number of EdU double-labelled *mbp*:GFP^+^ cells was also strongly reduced by 94% (Fig. 3E-G).

Importantly, we did not observe any *olig2*:GFP/*sox10*:mRFP double-labelled cells that were labelled by the TUNEL reaction, indicating that lower numbers of oligodendrocyte-lineage cells were not due to increased cell death (Fig. S3). To determine whether a sub-class of pMN progenitors show selective cell death, we also assessed the number of *olig2*:GFP/TUNEL double-labelled cells within the pMN domain, but did not detect increased cell death after a lesion. Occasionally, we observed double-labelled cells in the pMN domain outside the area of interest in both conditions, demonstrating that we were able to detect such cells (not shown). Taken together, these observations suggest that after a lesion, progenitor cells in the pMN domain switch from oligodendrocyte generation to motor neuron generation.

To elucidate whether other progenitor cells are induced to generate new neurons after a lesion, we analysed the number of newly generated dorsal *pax2a*:GFP^+^ interneurons, which are regenerated after an adult spinal cord lesion (Kuscha et al., 2012b). *pax2a*:GFP^+^ post-mitotic neurons have been suggested to be derived from more dorsal progenitor domains and not the pMN domain (England et al., 2011). The analysis in larvae showed equally low numbers of *pax2a*:GFP/EdU double-labelled cells in unlesioned controls and lesioned larvae (Fig. S4). This suggests that for some dorsal progenitor domains, a lesion might not be sufficient to trigger increased generation of neurons.

**Fig. 4.**
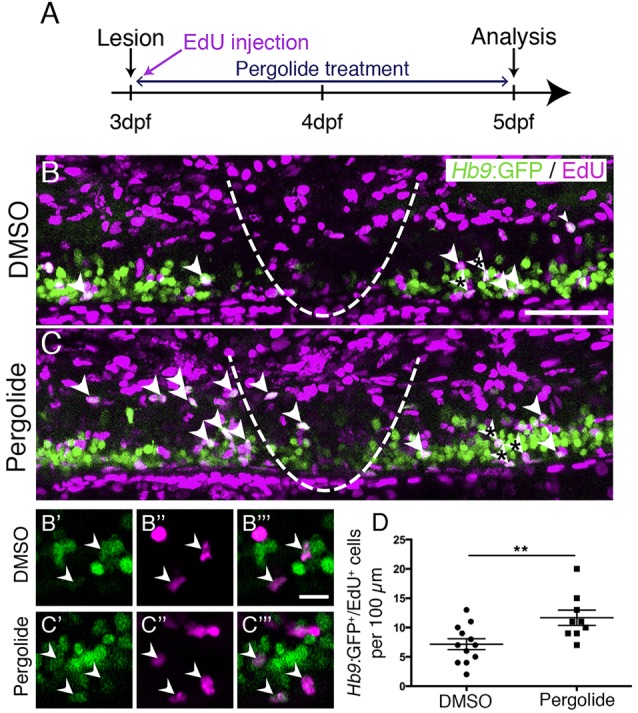
**Motor neuron regeneration is enhanced by application of a dopamine agonist.** (A) Experimental time line. (B,C) Double-labelled *Hb9:*GFP^+^/EdU^+^ neurons are indicated by arrowheads. (B′-C‴) Double-labelled cells from B,C (asterisks) are indicated by arrowheads in single optical sections at higher magnification. (D) Pergolide treatment during the regeneration phase significantly increases the number of *Hb9:*GFP^+^/EdU^+^ double-labelled motor neurons (*t*-test, **P=0.0092; DMSO, *n*=12; Pergolide, *n*=9). Lateral views are shown; rostral is left, dorsal is up. The lesion site is indicated by a dashed line. Values are means±s.e.m. Scale bars: 50 µm in B for B,C; 10 µm in B‴ for B′-C‴.

To determine whether larval regeneration shares mechanisms with adult regeneration, we decided to analyse dopamine signalling, which is shown to promote spinal motor neuron regeneration in adults (Reimer et al., 2013). To detect an endogenous source for dopamine, we labelled descending, mostly dopaminergic axons, by immunohistochemistry for tyrosine hydroxylase 1 (TH1; the rate-limiting enzyme in dopamine synthesis). Thus, we could detect descending TH1^+^ axons in the spinal cord of unlesioned larval zebrafish at 78 hpf (Fig. S5). In lesioned larvae, TH1^+^ axons were not detected caudal to the lesion site, 6 h after the lesion. These distal axon segments had most likely degenerated. Incubation in the dopamine agonist pergolide after a lesion (3-5 dpf), increased the number of newly generated *Hb9*:GFP^+^ motor neurons by 63% (Fig. 4), with no difference between the rostral and caudal spinal cord. These data suggest that larval pMN progenitors react to similar signals as adult pMN-like progenitors after a lesion.

### The immune response is important for motor neuron regeneration

**Fig. 5.**
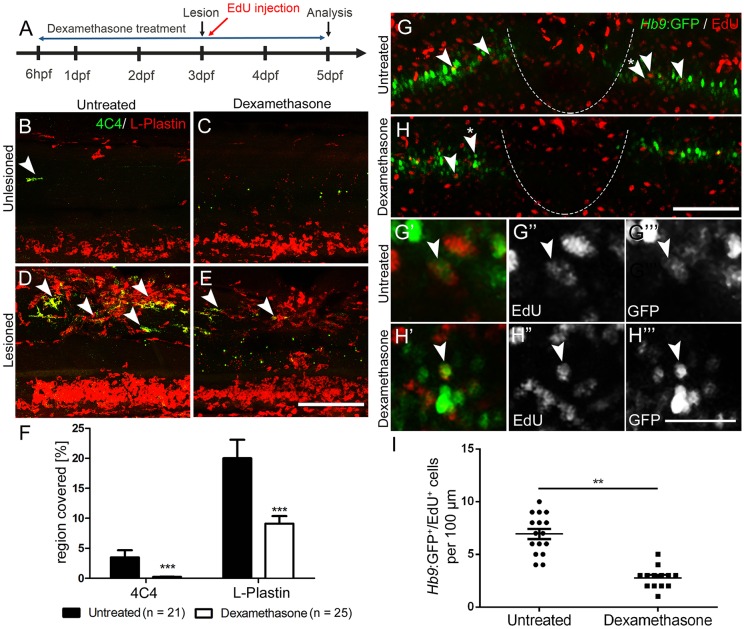
**Suppression of the immune response inhibits motor neuron regeneration.** (A) Time line for the experiments. (B-F) Incubation with dexamethasone does not lead to visible changes in unlesioned larvae (B,C), but strongly reduces the immune reaction at the lesion site (D,E) as indicated by reduced 4C4 (arrowheads indicate 4C4^+^ cells) and L-plastin immunoreactivity. Quantification of immunoreactivity is shown in F (Student's *t*-test, ***P<0.001). (G-I) Dexamethasone treatment reduces the number EdU-labelled *Hb9:*GFP^+^ motor neurons (arrowheads). Higher magnifications of double-labelled neurons indicated by asterisks in G,H are shown in single optical sections in G′-H‴. (I) Quantification of the reduction in newly generated motor neurons (*t*-test; **P=0.0085; unlesioned, *n*=16; dexamethasone, *n*=13). Lateral views are shown; rostral is left, dorsal is up. The lesion site is indicated by a dashed line. Values are means±s.e.m. Scale bars: 100 µm in E for B-E; 100 µm in H for G,H; 50 µm in H‴ for G′-H‴.

Reactive immune cells promote neuronal regeneration (Kyritsis et al., 2012) and the larval spinal cord offers the opportunity to study the innate immune system in isolation from the adaptive immune response, which develops only later (Danilova and Steiner, 2002; Lam et al., 2004). To determine whether the innate immune system promoted motor neuron generation after a larval lesion, we labelled microglial cells with a 4C4 antibody (Becker and Becker, 2001), and all macrophages and neutrophils with an L-plastin (Lcp1) antibody (Feng et al., 2010). Labelling with the 4C4 antibody was significantly increased in the lesion site by 369% (data not shown). At 48 h after the lesion, labelling of both 4C4 and L-plastin was concentrated at the lesion site (Fig. 5A,B,D). Suppressing this response by incubation of larvae in the immunosuppressant dexamethasone (Kyritsis et al., 2012), strongly dampened the microglia/macrophage response (Fig. 5C,E,F). This treatment also led to a reduction in the number of newly generated *Hb9*:GFP^+^/EdU^+^ motor neurons by 60% within 48 h of the lesion (3-5 dpf; Fig. 5G-I), suggesting that macrophages/microglial cells exert a positive influence on motor neuron regeneration.

### Genetic ablation of motor neurons triggers specific motor neuron apoptosis and macrophage/microglial activation

**Fig. 6.**
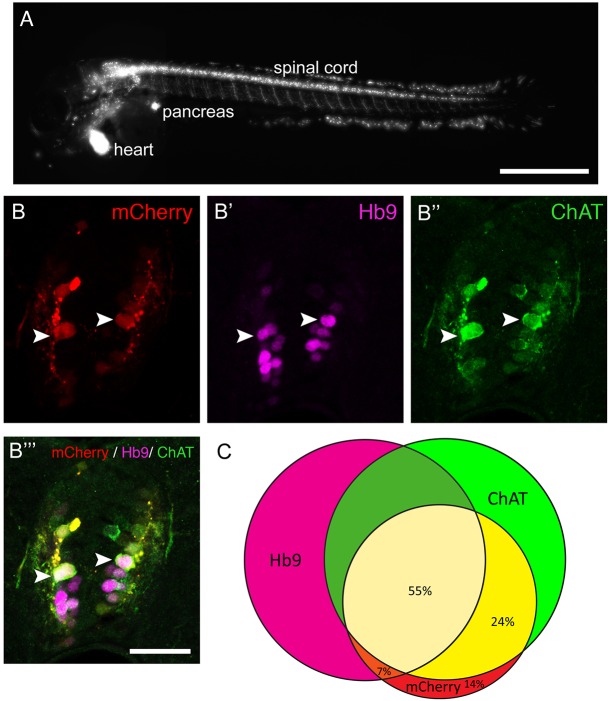
**The *Tg(mnx1:Gal4, UAS:nfsB-mCherry)* transgene is expressed in motor neurons.** (A) Lateral view of a whole larva (rostral left, dorsal up, 3 dpf) indicates labelling in spinal motor neurons, the pancreas and heart. (B,C) Spinal cross sections (3 dpf) indicate that most mCherry^+^ cells are also Hb9^+^ or ChAT^+^, or both. Arrowheads indicate triple-labelled cells. (C) Venn diagram showing the overlap of mCherry expression with motor neuron markers. Scale bars: 500 µm in A; 25 µm in B‴ for B-B‴.

To determine whether a mechanical lesion is necessary to trigger motor neuron regeneration or whether loss of motor neurons alone is sufficient to trigger a regenerative response, we expressed a nitroreductase transgene in motor neurons for selective ablation in *Tg(mnx1:Gal4, UAS:nfsB****-****mCherry)* double-transgenic fish. Lateral views of larvae showed obvious labelling in motor neurons in the ventral half of the spinal cord with axons growing into the muscle periphery, as well as in the heart and pancreas at 3 dpf (Fig. 6A). Expression levels varied, but could be substantially increased through selective breeding. There was a general decline in the number of labelled cells with progressing development. mCherry^+^ cells were still detectable at 10 dpf, but had completely disappeared in adults (data not shown).

At 3 dpf, triple labelling of Hb9, ChAT and mCherry revealed that 86% of the mCherry-labelled spinal cells were also positive for Hb9, ChAT or both, indicating that the vast majority of mCherry^+^ cells were indeed motor neurons (Fig. 6B,C). Conversely, 42% of Hb9^+^ and 54% of ChAT^+^ motor neurons expressed the *nfsB-mCherry* transgene at 3 dpf (Table S1). At 5 dpf, the proportion of Hb9^+^ motor neurons that were also labelled by mCherry was reduced to 25% (data not shown), in line with the developmental reduction in transgene and endogenous *hb9* expression.

**Fig. 7.**
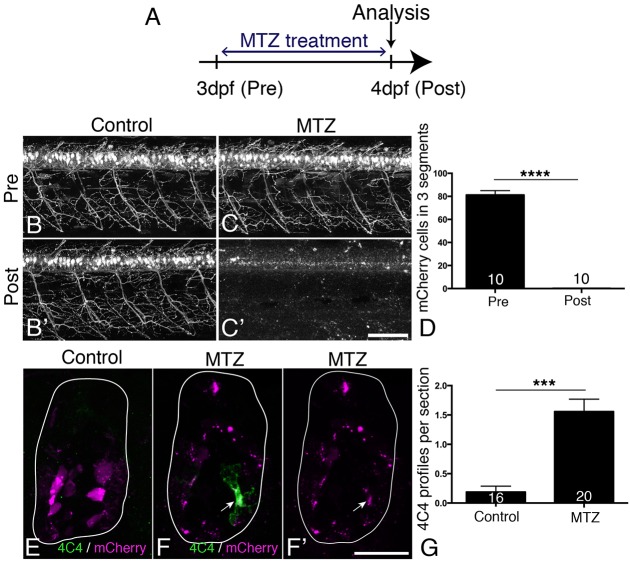
**MTZ treatment leads to ablation of all transgene-expressing cells and to microglia/macrophage activation.** (A) Treatment time line. (B-D) mCherry^+^ motor neurons and their axons are visible in untreated control larvae at 3 and 4 dpf (B,B′), but mCherry labelling is completely lost after 24 h treatment with MTZ (C,C′), quantified in D (Mann–Whitney *U*-test, ****P<0.0001). (E-G) Cross sections show that mCherry-labelled cells fragment during MTZ treatment and that microglia/macrophages appear and phagocytose the cell debris (arrow in F,F′). Microglia/macrophages are quantified in G (Mann–Whitney *U*-test, ***P<0.0001). Values are means±s.e.m. Scale bars: 100 µm in C′ for B-C′; 25 µm in F′ for E-F′.

**Fig. 8.**
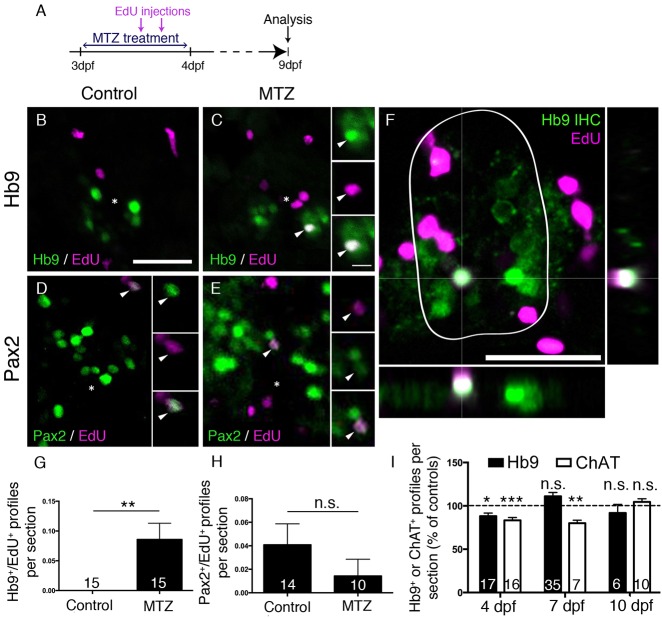
**Motor neuron ablation leads to regeneration of motor neurons.** (A) Experimental timeline. (B-H) In spinal cross sections, Hb9^+^/EdU^+^ motor neurons are only observed in MTZ-treated larvae (B,C, shown in a whole cross section of the spinal cord including orthogonal views in F), whereas Pax2^+^ interneurons are labelled by EdU in untreated and MTZ-treated larvae, quantified in G (Mann–Whitney *U*-test, **P=0.0063) and H (Mann–Whitney *U*-test, *P*>0.99), respectively. (I) Overall numbers of Hb9^+^ and ChAT^+^ profiles are reduced after a lesion, but return to control values at 7 dpf (Hb9 only) or 10 dpf (ChAT) (*t*-test, *P=0.0188; **P=0.0012; ***P=0.0004). Values are means±s.e.m. Scale bars: 25 µm in B for B-E; 5 µm in inset in C for all insets; 25 µm in F.

Incubation with metronidazole (MTZ) led to a visible loss of mCherry signal starting 4-5 h into the treatment and by 24 h, almost no intact cell bodies were observable in the spinal cord of whole-mounted larvae or sections (Fig. 7A-D; Fig. S6). TUNEL and FLICA labelling of motor neurons confirmed loss of these cells (Fig. S7). In *Tg(mnx1:Gal4, UAS:nfsB****-****mCherry); slc6a5*:GFP triple-transgenic fish, in which glycinergic neurons in the ventral spinal cord are additionally labelled for GFP (McLean et al., 2007), mCherry^+^ motor neurons, but not *slc6a5*:GFP^+^ interneurons were ablated, indicating specificity of ablation (Fig. S8). Counts of Hb9 and ChAT immunoreactive neurons showed a reduction by 12% and 17%, respectively (Fig. 8I). Treatment of *Hb9:*GFP larvae with 5 and 10 mM MTZ did not result in any cell loss, indicating that MTZ alone was not toxic to motor neurons (data not shown).

mCherry fluorescence was also reduced in the heart (Fig. S6), suggesting ablation of heart tissue and blood flow in ventral and intersegmental vessels had stopped in at 24 h into the treatment (*n*=5), but not in MTZ-treated control animals (*n*=5). These larvae still survived the treatment for several days. This is probably because small zebrafish larvae do not depend on circulation for oxygen diffusion until about 14 dpf (Jacob et al., 2002). For example, mutants without a heartbeat show no obvious aberrations in the first week of development (Rottbauer et al., 2001). The latest time point we analysed was at 9 dpf.

Using the 4C4 antibody, we detected that MTZ treatment resulted in an increased number of 4C4^+^ cell profiles in the spinal cord, which were often found to contain red fluorescing debris from mCherry^+^ cells after 24 h of MTZ incubation (Fig. 7E-G). The increase in numbers of macrophages/microglial cells was specific to the spinal cord, because we did not observe an increase in cell numbers in the eyes or brain (Fig. S9). Moreover, in wild-type larvae, no increase in macrophage/microglial cell counts was observed after MTZ treatment (data not shown). Hence, ablation of motor neurons leads to a specific reaction of macrophages/microglial cells in the spinal cord.

### Motor neurons regenerate after targeted ablation

As ablation of mCherry^+^ cells in *Tg(mnx1:Gal4, UAS:nfsB****-****mCherry)* double transgenic fish was almost complete, reappearance of these cells would indicate regeneration of motor neurons. However, we did not observe any new mCherry^+^ cells for at least 7 days post-ablation (*n*=7). To test whether this was due to a lack of regeneration or an inability to re-express the transgene, we treated larvae with MTZ from 3 to 4 dpf and additionally inflicted a mechanical lesion at 4 dpf, because mechanical lesions trigger motor neuron regeneration (see above). However, no mCherry^+^ cells were observed for up to 8 dpf (*n*=7, data not shown). In addition, at 2 weeks after a spinal lesion in adult *Tg(mnx1:Gal4, UAS:nfsB****-****mCherry)* fish (*n*=3), we observed small strongly Hb9 immunoreactive cells, previously shown to be lesion-induced newly generated motor neurons (Reimer et al., 2008), but none of these were mCherry^+^ (data not shown). This indicates that the transgene is not expressed by newly generated motor neurons at later stages of development or in adult fish.

To determine whether motor neuron regeneration took place after ablation of motor neurons, we used Hb9 immunohistochemistry after EdU injections at different injection and detection time points. After two injections of EdU at 16 and 22 h into the MTZ treatment, we observed a significant increase in the number of Hb9^+^/EdU^+^ motor neurons at 9 dpf (Fig. 8A-C,F,G) but not at 5 dpf (48 h after treatment onset; *n*=4, data not shown). By contrast, the number of Pax2 immunoreactive interneurons that were double labelled with EdU was not increased at 9 dpf after motor neuron ablation at 3 dpf (Fig. 8D,E,H). Application of EdU at 48 and 72 h post treatment did not result in Hb9/EdU co-labelled profiles in either controls (*n*=4) or MTZ-treated larvae (*n*=4 at 48 h post-treatment; *n*=4 at 72 h post treatment), indicating that new motor neurons were generated within 48 h of treatment.

The overall number of Hb9^+^ profiles was significantly reduced 24 h after ablation at 3 dpf and was back to control levels at 7 dpf. Similarly, numbers of ChAT profiles were significantly reduced up to 7 dpf and were not significantly different from controls by 10 dpf (Fig. 8I). This suggests that at least some newly generated motor neurons mature into ChAT-positive cells by 10 dpf. Taken together, the data suggest that motor neuron ablation leads to a rapid lesion-induced regeneration of motor neurons, which is completed within 48 h of ablation, followed by slow differentiation of motor neurons, whereas generation of Pax2^+^ interneurons was unaffected by motor neuron ablation.

## DISCUSSION

Here, we demonstrate that spinal progenitors can be induced to regenerate motor neurons in larval zebrafish at the expense of oligodendrocyte generation and identify the immune response as a regeneration-promoting signal. This supports the view that spinal progenitors in zebrafish are highly plastic in terms of the developmental programs they execute. Moreover, we show that genetic ablation of a proportion of motor neurons is sufficient to induce their specific regeneration.

### Larval motor neuron regeneration is similar to adult regeneration

Our previous birth dating study of motor neurons indicated that motor neuron generation sharply declines at 48 hpf (Reimer et al., 2013). Our present results confirm that few motor neurons are generated after that time. This allows us to distinguish regenerative from developmental neurogenesis at early larval stages. However, the larval zebrafish spinal cord is still a developing system. This is demonstrated by the observation that Pax2^+^ interneurons (this report) and other interneurons (Briona and Dorsky, 2014) are still being generated. Nevertheless, some key features of motor neuron regeneration are similar between larval and adult regeneration. For example, the highest numbers of regenerating motor neurons are observed close to the lesion site, motor neurons are derived from *olig2*-expressing progenitors and regeneration is promoted by dopamine (this study and Reimer et al., 2008; Reimer et al., 2013). We show here that motor neuron regeneration and functional recovery in larvae are very rapid, occurring within 48 h, whereas in adults, motor neuron regeneration and recovery of swimming take weeks. However, the possible contribution of regenerated motor neurons to functional recovery still needs to be determined. Our observations show that motor neuron regeneration can be studied in larvae.

### Microglia/macrophage signalling might contribute to regeneration

Another aspect that is similar for larval and adult lesions (Becker and Becker, 2001) is activation of the immune system, which might carry pro-regenerative signals. Here, we demonstrate accumulation of immune cells at a spinal lesion site and after motor neuron ablation in larvae and we find that suppression of the immune response using dexamethasone reduces motor neuron regeneration. Manipulation of the adult immune response with dexamethasone in the stab-lesioned telencephalon similarly suppressed neurogenesis from progenitor cells (Kyritsis et al., 2012). Analysis of the immune response at early larval stages has the advantage that the innate immune system can be studied in isolation of the adaptive immune system, because the latter is not functional at early larval stages (Danilova and Steiner, 2002; Lam et al., 2004). Hence, microglia/macrophages are positive regulators of lesion-induced neurogenesis in the lesioned adult and larval CNS.

### pMN progenitors are highly plastic

It is likely that regenerated motor neurons are derived from the pMN domain, because they are found close to it in the ventral spinal cord, the pMN domain exhibits increased proliferation after a lesion and new motor neurons retain DsRed protein expressed from the *olig2* promoter. The pMN progenitor domain generates motor neurons during embryonic development (Shin et al., 2007) and can be reactivated to generate motor neurons from relative quiescence in adults (Reimer et al., 2008). Here, we demonstrate that motor neuron generation can be reactivated by either transection or ablation lesion, even when pMN progenitors are actively generating oligodendrocytes at larval stages (Park et al., 2005; Czopka et al., 2013).

During development, evidence suggests that distinct pMN progenitors generate either motor neurons or oligodendrocytes in a time-dependent fashion (Wu et al., 2006; Ravanelli and Appel, 2015). In the context of larval regeneration, this means that either the oligodendrocyte-restricted progenitors change their developmental program to generate motor neurons, or that new motor neuron progenitors are recruited after lesion/ablation. Our observation that oligodendrogenesis sharply declines during motor neuron regeneration supports a view in which pMN progenitors change fate from oligodendrogenesis to motor neuron generation after a lesion.

### Ablated motor neurons are slowly regenerated

Ablation of a specific cell type allows us to ask whether the loss of this cell type is sufficient to elicit its regeneration. We found that after ablation of motor neurons, these are replenished over the course of a few days. Cell numbers for the immature motor neuron marker Hb9 were back to control levels earlier than those for the mature motor neuron marker ChAT. This reflects the differentiation sequence in developing motor neurons and in adult regeneration (Reimer et al., 2008). Interestingly, we observed new motor neurons (Hb9^+^/EdU^+^) by 48 h after the transection lesion, whereas after ablation, these were only observed at later time points. However, new motor neurons could no longer be labelled by EdU application at 48 h or later after the onset of ablation. This suggests rapid generation of new neuroblasts after ablation, followed by a prolonged differentiation phase. Interestingly, in mice, astrocytes in the telencephalon express a neurosphere-forming potential *in vitro* after a mechanical stab injury, but not after cell ablation, indicating that in mammals too, a mechanical lesion might lead to a stronger regenerative response in glial cells (Sirko et al., 2013).

In general, motor neuron regeneration in larvae is substantially quicker than lesion-induced regeneration in adults, in which newly generated, mature ChAT^+^ motor neurons were observed at 42 days, but not 14 days post injury (Reimer et al., 2008). Rapid motor neuron regeneration is an advantage of larval regeneration studies.

### Progenitor domains differ in their regenerative potential

Generation of *pax2a*:GFP^+^ or Pax2^+^ neurons was not enhanced in either of the experimental paradigms. These cells are most likely derived from a more dorsal progenitor domain than the pMN domain (England et al., 2011), indicating that progenitors for dorsal Pax2^+^ cells do not react to either a mechanical spinal lesion or ablation of motor neurons with enhanced generation of Pax2^+^ neurons. This underscores the highly plastic nature of the pMN progenitor domain.

### Technical considerations of genetic motor neuron ablation

The *Tg(mnx1:Gal4, UAS:nfsB-mCherry)* transgene is reliably and specifically expressed in a proportion of motor neurons at 3 dpf. Ablation is complete, with undetectable bystander effects on *slc6a5*:GFP^+^ interneurons. This all-or-nothing situation could be harnessed for regeneration screens in the future. However, even though Hb9^+^ motor neurons regenerate, the transgene, driven by *hb9* regulatory sequences, is not re-expressed. Possible explanations are developmentally reduced expression of the endogenous gene (Reimer et al., 2008) and silencing of the highly repetitive UAS sequences used in the generation of this transgenic model (Akitake et al., 2011).

Motor neuron ablation in this system leads to an impaired swimming behaviour (Reimer et al., 2013). This could become interesting for future studies of behavioural recovery during motor neuron regeneration. Currently, oedema formation due to ablation of heart cells precludes behaviour studies. Finally, we cannot exclude the possibility that the cessation of blood flow affects regeneration dynamics, even though larvae do not depend on oxygen (Jacob et al., 2002) and mutants without circulation develop normally for at least a week (Rottbauer et al., 2001). Next-generation models might employ less-repetitive versions of UAS (Akitake et al., 2011), which could be driven by regulatory sequences of more mature motor neuron markers (e.g. ChAT). Hence, ablation of motor neurons holds promise for future screening and functional studies of regeneration.

### Conclusion

Motor neuron progenitor cells can be induced to regenerate motor neurons at larval stages by mechanical lesion or motor neuron ablation and they react to similar signals (e.g. dopamine) as progenitor cells do during adult regeneration. This indicates considerable plasticity of larval progenitor/stem cells. We use this paradigm to demonstrate a pro-regenerative role of the immune system. Of note, some experiments can be performed at very early unprotected larval stages, thus replacing the need for work with older animals in the sense of reduction, refinement and replacement (the 3Rs; Tannenbaum and Bennett, 2015). We expect that the combination of rapid regeneration with the classical advantages of the zebrafish larva, i.e. small size, transparency and genetic accessibility will lead to a wide range of larval regeneration studies.

## MATERIALS AND METHODS

### Animals

All fish were kept and bred in our laboratory fish facility according to standard methods (Westerfield, 2000) and all experiments were approved by the British Home Office. We used wild-type (*wik*); *Tg(mnx1:GFP^ml2^)*, abbreviated as *Hb9:*GFP (Flanagan-Steet et al., 2005); *Tg(slc6a5:GFP)*, abbreviated as *slc6a5*:GFP (McLean et al., 2007); *Tg(mnx1:Gal4^s300t^)* (Wyart et al., 2009); *Tg(UAS:nfsB-mCherry)* (Davison et al., 2007); *Tg(olig2:EGFP)*, abbreviated as *olig2*:GFP (Shin et al., 2003); *Tg(olig2:DsRed2)*, abbreviated as *olig2*:DsRed (Kucenas et al., 2008); *Tg(pax2a:GFP)*, abbreviated as *pax2a*:GFP (Picker et al., 2002); *Tg(sox10(7.2):mRFP)*, abbreviated as *sox10*:mRFP (Kirby et al., 2006); and *Tg(mbp:EGFP)*, abbreviated as *mbp*:GFP (Almeida et al., 2011). Male and female fish were used for the experiments.

### Larval lesion

Larvae were anaesthetized in 0.02% MS222 (Sigma-Aldrich), embedded in 1.5% low melting point agarose and placed in a lateral position on a microscope slide. Lesions were performed using a sharp 30-gauge injection needle, leaving the notochord and major blood vessels intact. After the lesions, larvae were either immediately injected with 5-ethynyl-2′-deoxyuridine (EdU; Sigma-Aldrich) into the yolk (as described below) or immediately released by gently removing the agarose.

### Quantification of behavioural recovery

Lesioned and unlesioned control larvae were touched with a glass capillary on the median fin fold caudal to the lesion site. The swim path of their escape response was recorded and analysed using a Noldus behaviour analysis set-up and EthoVision software (v. 7). Data are shown as distance travelled during the first 15 s after touch, averaged from triplicate measures per larva. Completeness of injury was verified by the absence of an escape response in freshly lesioned larvae. Dependence of recovery on continuity of the spinal cord was tested by re-lesioning at the end of the regeneration period.

### Dexamethasone treatment

To suppress the immune system, we used the glucocorticoid receptor agonist dexamethasone (Sigma), which was previously shown to suppress the immune response in adults (Kyritsis et al., 2012) and larvae (Zhang et al., 2008; Hall et al., 2014). Embryos were incubated with 200 μg/ml dexamethasone in fish water containing 1% DMSO (Sigma-Aldrich) from 6 hpf. Embryos were kept in 5 ml Petri dishes in groups of 10 and fish water was changed every day. At 3 dpf, larvae were lesioned as described above and returned into clean Petri dishes with dexamethasone until analysis at day 5.

### Genetic ablation

For motor neuron ablation, larvae were treated from 72 hpf onwards for 24 h with 5 mM metronidazole (Sigma-Aldrich) in fish water containing 0.2% DMSO in darkness at 28°C.

### TUNEL labelling

Cryosections were dehydrated in methanol for 10 min at −20°C, washed for 10 min in PBS at room temperature. Terminal deoxynucleotidyl transferase dUTP nick end labelling (TUNEL) was performed using the Fluorescein In Situ Cell Death Detection Kit (Roche) according to the manufacturer's protocol. Sections were covered with Parafilm to prevent evaporation and incubated in a humid chamber at 37°C for 1 h. After the labelling procedure, the sections were extensively washed in PBS.

Whole mounted zebrafish larvae at 5 days post lesion were anaesthetized, fixed in 4% paraformaldehyde (PFA)/1% DMSO in PBS at room temperature for 3 h, washed in PBS and PBST (PBS with 0.2% Tween 20). Then larvae were digested with collagenase (Sigma; 0.2 mg/ml in PBST) for 50 min. The TDT reaction cocktail was prepared according to the manufacturer's instructions (Click-iT TUNEL imaging assay; Roche) and larvae were incubated at room temperature overnight. Larvae were washed in PBS and Click-iT reaction cocktail was prepared and larvae were incubated at room temperature in the dark for 3 h. After extensive washing in PBS, larvae were either processed for immunohistochemistry or transferred to 70% glycerol in PBS.

### Detection of activated caspases using fluorochrome-labelled inhibitors of caspases

Fluorochrome-labelled inhibitors of caspases (FLICAs) were used for the detection of activated caspases in motor neurons during metronidazole treatment. We adapted this method for *in vivo* zebrafish larvae from that recently reported for the *ex vivo* brain and spinal cord of larval sea lampreys (Barreiro-Iglesias and Shifman, 2012). The Image-iT LIVE Green Poly Caspases Detection Kit (Invitrogen) was used for the detection of activated caspases in motor neurons. This kit contains one vial (component A of the kit) of the lyophilized FLICA reagent (FAM-VAD-FMK). The reagent contains a fluoromethyl ketone (FMK) moiety, associated with a caspase-specific amino acid sequence (VAD). A carboxyfluorescein group (FAM) is attached as a fluorescent reporter. The FLICA reagent interacts with the enzyme active centre of any activated caspase via the recognition sequence, and then attaches covalently through the FMK moiety (Barreiro-Iglesias and Shifman, 2012). The 150× stock FLICA reagent solution was prepared by adding 50 µl of DMSO to the vial.

Six hours after treatment, DMSO control and metronidazole-treated 78 hpf *Tg(mnx1:Gal4, UAS:nfsB****-****mCherry)* zebrafish were incubated at 28.5°C in 1× FLICA labelling solution (made from 150× FLICA reagent stock diluted in PBS) for 1 h. After incubation, the animals were washed in PBS on a shaker 9×15 min in PBS to remove unbound FLICA (Barreiro-Iglesias and Shifman, 2012). After washes, the animals were anaesthetized and fixed in 4% PFA in PBS for 1 h and then mounted in 70% glycerol in PBS.

### Antibodies

We used mouse anti-Hb9 (MNR2, Developmental Studies Hybridoma Bank, 1:400) (Reimer et al., 2013); rabbit anti-Pax2 (PRB-276P, Covance, 1:1000) (Kuscha et al., 2012b), goat anti-choline acetyltransferase (ChAT) (AB144P, Millipore, 1:500) (Reimer et al., 2008), rabbit anti-L-plastin (a gift from Yi Feng, University of Edinburgh; 1:500) recognizing macrophages, microglia and neutrophils (Feng et al., 2010) and the mouse monoclonal antibody 4C4 (hybridoma line 7.4.C4; 92092321, Public Health England; supernatant, 1:50), recognizing microglia/macrophages (Becker and Becker, 2001), as previously described.

### Whole-mount immunohistochemistry

Whole larvae were fixed in 4% PFA/1% DMSO in PBS for 2 h. After washing in PBS, larvae were permeabilized in collagenase (2 mg/ml in PBS) for 25 min. After washing with PBStx (0.2% Triton X-100 in PBS) larvae were incubated in 50 mM glycine for 10 min and washed. After blocking in blocking buffer (1% DMSO, 1% goat serum, 1% BSA and 0.7% Triton X-100) for 2 h, larvae were incubated with primary antibodies at 4°C overnight. Secondary antibodies were added after extensive washes at 4°C overnight. After washing, larvae were mounted in 70% glycerol for subsequent imaging.

### Section immunohistochemistry

Larvae were fixed in 4% paraformaldehyde for 2 h, cryoprotected in 30% sucrose overnight, flash-frozen and cryosectioned at a thickness of 14 µm. Sections were fixed in ice cold methanol at −20°C for 10 min and then rehydrated in PBS at room temperature for 10 min. After incubation in blocking buffer (2% goat serum in 0.2% Triton X-100 in PBS) at room temperature for 1.5 h they were incubated with primary antibody overnight at 4°C. Sections were washed in PBStx (0.2% Triton X-100 in PBS) before secondary antibody incubation for 1 h at room temperature. After three 5 min washes in PBStx and two 5 min washes in PBS, sections were mounted in Fluoromount (Sigma).

### EdU labelling and detection

Zebrafish larvae from 3 days post-lesion onwards were anaesthetized, placed on a silicone elastomere mount (Sylgard 527 A&B; Dow Corning, 1675167) and injected into the yolk with ∼5 nl 5 mM EdU in 0.1 M sterile KCl and 7.5% DMSO. After washing, larvae were allowed to develop under standard conditions. For analysis, larvae were fixed in 4% paraformaldehyde for 2 h, cryoprotected in 30% sucrose overnight, flash-frozen and cryosectioned at 14 µm. Sections were fixed in methanol for 10 min at −20°C and washed for 10 min in PBS at room temperature. The EdU Click-iT reaction solution (Roche) was prepared fresh according to the manufacturer's protocol. Sections on slides were covered with solution and incubated in a humid chamber at room temperature in the dark for 3 h. After three 10 min washes in PBS, sections were used for imaging or subsequent processing for immunohistochemistry.

Whole-mounted larvae were fixed in 4% PFA/1% DMSO in PBS at room temperature for 3 h, washed in PBS, transferred to methanol and incubated at −20°C for at least 2 h. After rehydrating in a dilution row from methanol to PBST (0.2% Tween 20) larvae were washed in PBST and digested with collagenase (Sigma; 0.2 mg/ml in PBST) for 30 min. The EdU Click-iT reaction solution was prepared and larvae were incubated in 2 ml tubes at room temperature in the dark for 3 h. After extensive washing in PBS, larvae were either processed for immunohistochemistry or transferred to 70% glycerol in PBS.

### Quantification and statistical analyses

Cell quantifications in whole-mounted larvae were performed manually by analysing all consecutive images in stacks taken with a confocal microscope. Multiply labelled cells were scored when labels occurred on the same optical section (<2 µm). As a standard, counts were performed within the 50 µm rostral and caudal closest to the lesion site and expressed as cells/100 µm, unless stated differently. For quantification in cryosections (14 µm thickness) we counted all cell profiles and expressed this as number of profiles/section. All variability indicated represents s.e.m. All images were captured with a 20× objective on an Zeiss LSM710 confocal microscope. At least two independent experiments were performed per treatment and cell counts were carried out blinded to the treatment. We used *t*-tests (for normally distributed data) and Mann–Whitney *U*-tests (for non-normal distributions) for comparisons of two conditions. For multiple comparisons, we used one-way analysis of variance followed by Bonferroni's test for comparisons of individual groups (for normally distributed data) or the Kruskal–Wallis test followed by Dunn's test for comparing individual groups (for non-normal distributions), using GraphPad Prism software.
